# Racial and ethnic disparities in gallbladder cancer: A two‐decade analysis of incidence and mortality rates in the US

**DOI:** 10.1002/cam4.7457

**Published:** 2024-07-04

**Authors:** Yazan Abboud, Lawanya Singh, Madison Fraser, Chun‐Wei Pan, Ibrahim Abboud, Islam H. Mohamed, David Kim, Saqr Alsakarneh, Fouad Jaber, Benjamin Richter, Ahmed Al‐Khazraji, Kaveh Hajifathalian, Sima Vossough‐Teehan

**Affiliations:** ^1^ Department of Internal Medicine Rutgers New Jersey Medical School Newark New Jersey USA; ^2^ Department of Internal Medicine John Stroger Hospital of Cook County Chicago Illinois USA; ^3^ University of California Riverside School of Medicine Riverside California USA; ^4^ Department of Internal Medicine University of Missouri‐Kansas City Kansas City Missouri USA; ^5^ Division of Gastroenterology and Hepatology Rutgers New Jersey Medical School Newark New Jersey USA

**Keywords:** disparities, gallbladder cancer, incidence, mortality, race

## Abstract

**Background:**

Gallbladder cancer (GBC) is an aggressive malignancy that is usually diagnosed at a late stage. Prior data showed increasing incidence of GBC in the US. However, little is known about race/ethnic‐specific incidence and mortality trends of GBC per stage at diagnosis. Therefore, we aimed to conduct a time‐trend analysis of GBC incidence and mortality rates categorized by race/ethnicity and stage‐at‐diagnosis.

**Methods:**

Age‐adjusted GBC incidence and mortality rates were calculated using SEER*Stat software from the United States Cancer Statistics database (covers ~98% of US population between 2001 and 2020) and NCHS (covers ~100% of the US population between 2000 and 2020) databases, respectively. Race/Ethnic groups were Non‐Hispanic‐White (NHW), Non‐Hispanic‐Black (NHB), Hispanic, Non‐Hispanic‐Asian/Pacific‐Islander (NHAPI), and Non‐Hispanic‐American‐Indian/Alaska‐Native (NHAIAN). Stage‐at‐diagnoses were all stages, early, regional, and distant stages. Joinpoint regression was used to generate time‐trends [annual percentage change (APC) and average APC (AAPC)] with parametric estimations and a two‐sided *t*‐test (*p*‐value cut‐off 0.05).

**Results:**

76,873 patients were diagnosed with GBC with decreasing incidence rates in all races/ethnicities except NHB who experienced an increasing trend between 2001 and 2014 (APC = 2.08, *p* < 0.01) and plateauing afterward (APC = −1.21, *p* = 0.31); (AAPC = 1.03, *p* = 0.03). Among early‐stage tumors (9927 patients), incidence rates were decreasing only in Hispanic (AAPC = −4.24, *p* = 0.006) while stable in other races/ethnicities (NHW: AAPC = −2.61, *p* = 0.39; NHB: AAPC = −1.73, *p* = 0.36). For regional‐stage tumors (29,690 patients), GBC incidence rates were decreasing only in NHW (AAPC = −1.61, *p* < 0.001) while stable in other races/ethnicities (NHB: AAPC = 0.73, *p* = 0.34; Hispanic: AAPC = −1.58, *p* = 0.24; NHAPI: AAPC = −1.22, *p* = 0.07). For distant‐stage tumors (31,735 patients), incidence rates were increasing in NHB (AAPC = 2.72, *p* < 0.001), decreasing in Hispanic (AAPC = −0.64, *p* = 0.04), and stable in NHW (AAPC = 0.07, *p* = 0.84) and NHAPI (AAPC = 0.79, *p* = 0.13). There were 43,411 deaths attributed to GBC with decreasing mortality rates in all races/ethnicities except NHB who experienced a stable trend (AAPC = 0.25, *p* = 0.25).

**Conclusion:**

Nationwide data over the last two decades show that NHB patients experienced increasing GBC incidence between 2001 and 2014 followed by stabilization of the rates. This increase was driven by late‐stage tumors and occurred in the first decade. NHB also experienced non‐improving GBC mortality, compared to other race and ethnic groups who had decreasing mortality. This can be due to lack of timely‐access to healthcare leading to delayed diagnosis and worse outcomes. Future studies are warranted to investigate contributions to the revealed racial and ethnic disparities, especially in NHB, to improve early detection.

## 1 INTRODUCTION

Gallbladder cancer (GBC) is an aggressive malignancy with a 5‐year survival rate of 20% in the US.^1^ According to data provided by the National Cancer Institute's Surveillance, Epidemiology, and End Surveillance program, only one in five gallbladder cancers are detected in early stages.^1^ This contributes to its lethality, given that most cases are identified only by the time of metastasis. Notably, even with surgical resection, median survival after late‐stage diagnosis of GBC ranges from 12 to 14 months.

The gallbladder is a small, pear‐shaped organ nestled beneath the liver—a concealed location lending to early GBC's ability to go undetectable. Due to the gallbladder's small size and location, early tumors are often undetectable on routine physical exam.^2^ The gallbladder's role is to store bile, a fluid that is secreted into the small intestine to aid in the digestion, absorption, and excretion of fats. Bile is synthesized in the liver and brought to the gallbladder for storage via the hepatobiliary tracts. During postprandial secretion, bile is expelled through the common bile duct, which joins with the pancreatic duct (carrying additional pancreatic enzymes important for digestion) and is secreted into the duodenum via the ampulla of Vater. The gallbladder contains four main layers of tissue: the mucosal inner layer (made up of secretory cells), muscular layer (aiding in the expulsion of bile), connective tissue layer, and serosal (outer) layer. The majority of GBCs are adenocarcinomas, originating from the mucosal inner layer and spreading outwards.^3^ Given that the gallbladder does not play a main role in producing bile, it is considered a non‐vital organ and can easily be removed when pathology arises. Thus, surgical intervention is the cornerstone of GBC management. This is essential to know especially given the disparities in access to surgical care and outcomes between different populations of the US.^4^

Well‐established risk factors and associations exist for GBC, including congenital malformations, autoimmune diseases (such as primary sclerosing cholangitis), Typhoid and *H. pylori* infections, obesity, and certain inflammatory markers such as C‐reactive protein (CRP).^5^ Most markedly, gallstone disease is a significant risk factor for later development of GBC, likely due to chronic inflammatory changes that occur in the gallbladder as a result. This leads to an observed increased incidence of GBC in females compared to males, due appreciably to estrogen's recognized association with predisposition to both gallstone disease and GBC. Additionally, genetics are thought to be responsible for up to a quarter of GBC cases. Certain ethnicities, such as Native Americans and Asians, are known to be at increased risk of GBC development.^6^

Prior data showed an increasing incidence of GBC in the United States with variations of trends between different race and ethnic groups. However, very little is known about recent race and ethnic‐specific incidence trends of early and late‐stage GBC. Therefore, the aim of the current study was to conduct a time‐trend analysis of GBC incidence rates categorized by stage at diagnosis and race/ethnic group, and of GBC mortality rates, with the goal of improving early GBC detection via identification of at‐risk ethnicities in the United States and evaluating their outcomes.

## 2 METHODS

This study is a nationwide time‐trend analysis of GBC incidence and mortality rates in the US between 2001–2020 and 2000–2020, respectively. Data used in this study were all public and de‐identified and therefore were exempted from institutional review board (IRB) review based on the National Human Research Protections Advisory Committee Policy.

Incidence rates of GBC between January 1, 2001, and December 31, 2020, were obtained from the United States Cancer Statistics (USCS) database, which is the most comprehensive source of cancer incidence data in the US, and nearly covers 98% of the population.^7^ Data included in the USCS database is compiled from the Centers for Disease Control and Prevention (CDC)'s National Program of Cancer Registries (NPCR) and the National Cancer Institute (NCI)'s Surveillance, Epidemiology, and End Results (SEER) program, covering 50 US states, the District of Columbia, and Puerto Rico. These data undergo standardized input and coding processes as per the North American Association of Central Cancer Registries' Data Standards to ensure maintaining high‐quality data.^8^

Mortality rates of GBC between 2000 and 2020 were obtained from the NCHS database, which is the most comprehensive source of mortality data in the US and nearly covers 100% of the population.^9^ Mortality data are compelled by the National Vital Statistics System which mainly uses an electronic death registration system. Death certificates are used to specify the cause of death based on the International Classification of Diseases (ICD). These data undergo standardized collection and monitoring processes to maintain high‐quality data.^9^

Incidence rate of GBC was specified as the number of patients diagnosed with GBC per 100,000 population each year. Mortality rate of GBC was specified as the number of deaths attributed to GBC per 100,000 population each year. Time‐trends were reported as annual percentage change (APC) and average APC (AAPC). Tumor location was specified as “Gallbladder” with malignant behavior. Stage at diagnosis was categorized into all stages, early stage (in situ and localized tumors) and late stage (tumors with regional or distant site/nodes involvement). Race/Ethnicity were categorized as Non‐Hispanic White (NHW), Non‐Hispanic Black (NHB), Hispanic, Non‐Hispanic Asian/Pacific Islander (NHAPI), and Non‐Hispanic American Indian/Alaska Native (NHAIAN).

Incidence rates of GBC were calculated and age‐adjusted to the standard 2000 US population using SEER*Stat software (v.8.4.1.2, National Cancer Institute “NCI”). APC and AAPC were computed using Joinpoint Regression Software (v.4.9.0.1, NCI) utilizing Monte Carlo permutation analysis to estimate the best‐fit trend that reflects the change of rates over time.^10^, ^11^ The trends were evaluated using parametric estimations utilizing a two‐sided *t*‐test and *p*‐value cut‐off of 0.05. The aforementioned analysis was performed in race/ethnic‐specific populations, and after categorizing the tumors by stage at diagnosis as specified above. Lastly, due to the potential impact of the COVID‐19 pandemic on diagnosing GBC, sensitivity analysis was conducted for race/ethnic‐specific incidence and mortality rates after excluding the year 2020.

## 3 RESULTS

### 3.1 Gallbladder cancer incidence rates and time‐trends in the USCS database

**FIGURE 1:**
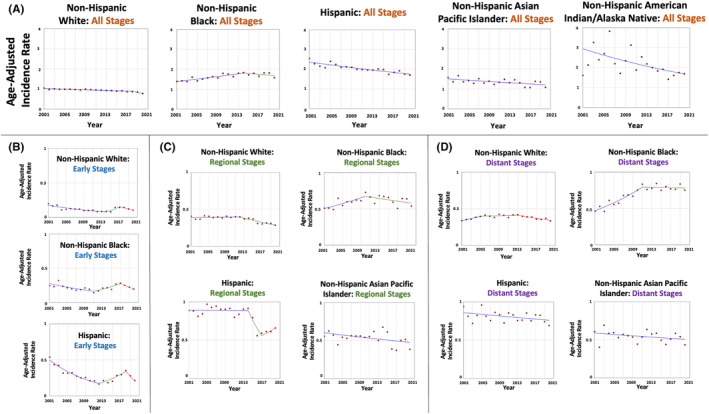
Time‐trends and age‐adjusted incidence rates per 100,000 population for gallbladder cancer among different race and ethnic groups categorized by tumor stage at diagnosis.

There were 76,873 patients who were diagnosed with GBC between 2001 and 2020. Overall, GBC age‐adjusted incidence rates per 100,000 population decreased in Hispanic from 2.54 in 2001 to 1.70 in 2020 (AAPC = −1.57, *p* < 0.001), NHAIAN from 1.62 in 2001 to 1.70 in 2020 (AAPC = −2.90, *p* = 0.004), and NHAPI from 1.56 in 2001 to 1.07 in 2020 (AAPC = −1.15, *p* = 0.01). For NHW, GBC incidence rates decreased from 1.05 in 2001 to 0.87 in 2018 (APC = −0.84, *p* < 0.001) and plateaued afterward (APC = −6.13, *p* = 0.06). However, GBC age‐adjusted incidence rates in NHB increased from 1.38 in 2001 to 1.83 in 2014 (APC = 2.08, *p* < 0.01) and plateaued afterward (APC = −1.21, *p* = 0.31) (Table 1 and Figure 1A). On average, the rates in NHB were increasing over the study period (AAPC = 1.03, *p* = 0.03).

**TABLE 1:** Time‐trends for gallbladder cancer incidence rates among different race and ethnic groups categorized by tumor stage at diagnosis.

Race/Ethnic group	Cases (*N* = 76,873)^a^	Trends^b^					
		Incidence rate in 2001	Time period	APC (95% CI)	*p*‐Value	AAPC (95% CI)	*p*‐Value
All stages							
NHW	48,777 (63.45%)	1.05	2001–2018	−0.84 (−1.06 to −0.61)	<0.001	−1.41 (−2.07 to −0.75)	<0.001
			2018–2020	−6.13 (−12.16 to 0.31)	0.06		
NHB	10, 648 (13.85%)	1.38	2001–2014	2.08 (1.14 to 3.03)	< 0.001	1.03 (0.10 to 1.97)	0.03
			2014–2020	−1.21 (−3.61 to 1.25)	0.31		
Hispanic	11, 101 (14.44%)	2.54	2001–2020	−1.57 (−1.94 to −1.20)	<0.001	−1.57 (−1.94 to −1.20)	<0.001
NHAPI	3646 (4.74%)	1.56	2001–2020	−1.15 (−1.99 to −0.30)	0.01	−1.15 (−1.99 to −0.30)	0.01
NHAIAN	790 (1.03%)	1.62	2001–2020	−2.90 (−4.68 to −1.07)	0.004	−2.90 (−4.68 to −1.07)	0.004
Early stage tumors							
NHW	6191 (8.05%)	0.20	2001–2014	−6.08 (−7.86 to −4.28)	<0.001	−2.61 (−8.30 to 3.44)	0.39
			2014–2017	25.75 (−13.82 to 83.49)	0.21		
			2017–2020	−11.71 (−25.82 to 5.08)	0.14		
NHB	1390 (1.81%)	0.24	2000–2011	−5.04 (−8.10 to −1.88)	0.005	−1.73 (−5.39 to 2.07)	0.36
			2011–2015	9.07 (0.08 to 18.88)	0.04		
			2015–2020	−10.58 (−24.77 to 6.29)	0.18		
Hispanic	1, 522 (1.98%)	0.54	2001–2012	−9.18 (−11.01 to −7.31)	<0.001	−4.24 (−7.12 to −1.26)	0.006
			2012–2018	12.01 (5.41 to 19.02)	0.002		
			2018–2020	−19.89 (−37.15 to 2.10)	0.07		
NHAPI	461 (0.60%)	0.20	*	*	*	*	*
NHAIAN	98 (0.13%)	*	*	*	*	*	*
Regional stage tumors							
NHW	18,999 (24.71%)	0.41	2001–2012	0.21 (−0.76 to 1.18)	0.65	−1.61 (−2.40 to −0.81)	<0.001
			2012–2020	−4.05 (−5.58 to −2.51)	<0.001		
NHB	3881 (5.05%)	0.51	2001–2010	3.27 (0.51 to 6.11)	0.02	0.73 (−0.77 to 2.25)	0.34
			2010–2020	−1.50 (−3.36 to 0.39)	0.11		
Hispanic	4316 (5.61%)	0.89	2001–2014	−0.04 (−0.99 to 0.91)	0.92	−1.58 (−4.19 to 1.09)	0.24
			2014–2017	−14.28 (−27.48 to 1.33)	0.06		
			2017–2020	5.63 (−2.29 to 14.19)	0.15		
NHAPI	1435 (1.87%)	0.55	2001–2020	−1.22 (−2.58 to 0.15)	0.07	−1.22 (−2.59 to 0.15)	0.07
NHAIAN	309 (0.41%)	*	*	*	*	*	*

Distant stage tumors							
NHW	19,911 (25.90%)	0.34	2001–2009	2.52 (0.96 to 4.10)	0.004	0.07 (−0.68 to 0.83)	0.84
			2009–2020	−1.67 (−2.52 to −0.80)	0.001		
NHB	4659 (6.06%)	0.94	2001–2011	5.41 (3.73 to 7.12)	<0.001	2.72 (1.71 to 3.75)	<0.001
			2011–2020	−0.18 (−1.57 to 1.23)	0.78		
Hispanic	4572 (5.95%)	0.46	2001–2020	−0.64 (−1.28 to −0.00)	0.04	−0.64 (−1.28 to −0.00)	0.04
NHAPI	1520 (1.98%)	0.61	2001–2020	−0.79 (−1.82 to 0.26)	0.13	−0.79 (−1.82 to 0.26)	0.13
NHAIAN	310 (0.40%)	*	*	*	*	*	*

Abbreviations: NHW, Non‐Hispanic White; NHB, Non‐Hispanic Black; NHAPI, Non‐Hispanic Asian/Pacific Islander; NHAIAN, Non‐Hispanic American Indian/Alaska Native.

^a^ Data are presented as death numbers followed by percentages of the total deaths of gallbladder cancer in the database.

^b^ Time‐trends were computed using Joinpoint Regression Program (v4.9.0.1, NCI) with 3 maximum joinpoints allowed (4‐line segments).

* There were too few cases of gallbladder cancer at least in one calendar year to estimate rates or trends.

When evaluating the tumors stratified by stage at diagnosis, the trends varied. In early‐stage tumors (9927 patients; 12.9% of all cases), GBC age‐adjusted incidence rates per 100,000 population decreased only in Hispanics from 0.54 in 2001 to 0.22 in 2020 (AAPC = −4.24, *p* = 0.006). The rates were stable in NHW (AAPC = −2.61, *p* = 0.39) and NHB (AAPC = −1.73, *p* = 0.36) (Table 1 and Figure 1B). There were too few cases in NHAPI and NHAIAN hindering the estimation of a trend.

For regional‐stage tumors (29,690 patients; 38.6% of all cases), GBC age‐adjusted incidence rates per 100,000 population in NHW were stable between 2001–2012 and decreased afterward; on average, the rates decreased from 0.41 in 2001 to 0.28 in 2020 (AAPC = −1.61, *p* < 0.001). The rates were stable in the other racial and ethnic groups; NHB (AAPC = 0.73, *p* = 0.34), Hispanic (AAPC = −1.58, *p* = 0.24), and NHAPI (AAPC = −1.22, *p* = 0.07) (Table 1 and Figure 1C). There were too few cases in NHAPI hindering the estimation of a trend.

For distant‐stage tumors (31,735 patients; 41.3% of all cases), there was a variation between the trends. GBC age‐adjusted incidence rates per 100,000 population increased in NHB from 0.46 in 2001 to 0.83 in 2011 (APC = 5.41, *p* < 0.001) and plateaued afterward (APC = −0.18, *p* = 0.78). On average, the rates in NHB increased over the study period (AAPC = 2.72, *p* < 0.001). In Hispanic, GBC incidence rates decreased from 0.94 in 2001 to 0.69 in 2020 (AAPC = −0.64, *p* = 0.04) (Table 1 and Figure 1D). The rates were stable in NHW (AAPC = 0.07, *p* = 0.84) and NHAPI (AAPC = ‐0.79, *p* = 0.13). There were too few cases in NHAPI hindering the estimation of a trend.

### 3.2 Gallbladder cancer mortality rates and time‐trends in the NCHS database

**FIGURE 2:**
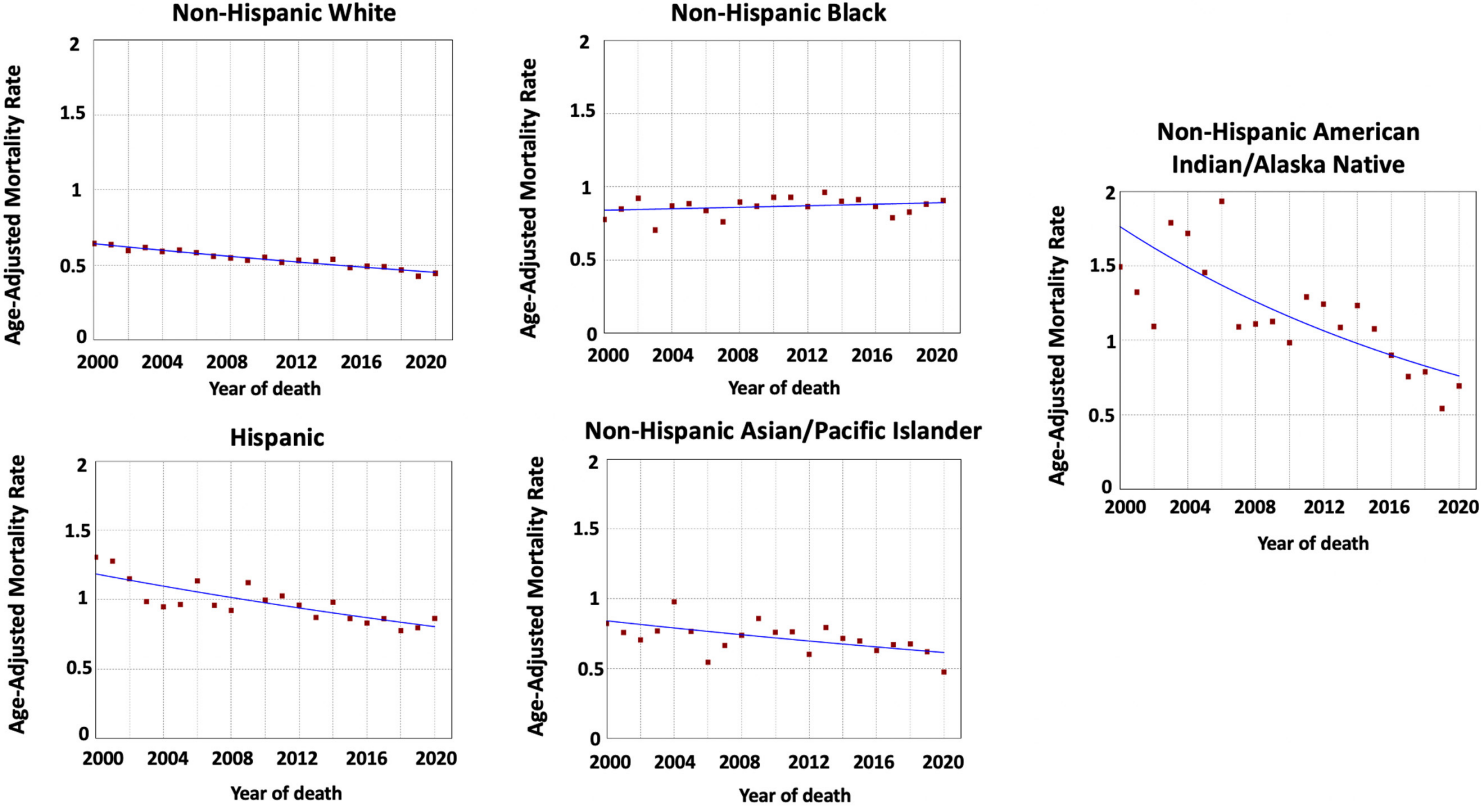
Time‐trends and age‐adjusted mortality rates per 100,000 population for gallbladder cancer among different race and ethnic groups.

There were 43,411 deaths attributed to GBC in the US between 2000 and 2020. Overall, GBC age‐adjusted mortality rates per 100,000 population decreased in NHAIAN from 1.49 in 2000 to 0.69 in 2020 (AAPC = −4.12, *p* < 0.001), Hispanic from 1.31 in 2000 to 0.86 in 2020 (AAPC = −1.92, *p* < 0.001), NHAPI from 0.82 in 2000 to 0.47 in 2020 (AAPC = −1.56, *p* = 0.004), and NHW from 0.64 in 2000 to 0.44 in 2020 (AAPC = −1.82, *p* < 0.001) (Table 2 and Figure 2). However, the rates were stable in NHB (AAPC = 0.25, *p* = 0.25).

**TABLE 2:** Time‐trends for gallbladder cancer mortality rates among different race and ethnic groups.

Race/Ethnic group	Deaths (*N* = 43,411)^a^	Trends^b^					
		Mortality rate in 2000	Time period	APC (95% CI)	*p*‐Value	AAPC (95% CI)	*p*‐Value
All stages							
NHW	29,981 (69.06%)	0.64	2000–2020	−1.82 (−2.05 to −1.59)	<0.001	−1.82 (−2.05 to −1.59)	<0.001
NHB	5725 (13.19%)	0.78	2000–2020	0.30 (−0.23 to 0.84)	0.25	0.30 (−0.23 to 0.84)	0.25
Hispanic	5268 (12.14%)	1.31	2000–2020	−1.92 (−2.50 to −1.33)	<0.001	−1.92 (−2.50 to −1.33)	<0.001
NHAPI	1938 (4.46)	0.82	2000–2020	−1.56 (−2.56 to −0.55)	0.004	−1.56 (−2.56 to −0.55)	0.004
NHAIAN	416 (0.96%)	1.49	2000–2020	−4.12 (−5.82 to −2.37)	<0.001	−4.12 (−5.82 to −2.37)	<0.001

Abbreviations: NHW, Non‐Hispanic White; NHB, Non‐Hispanic Black; NHAPI, Non‐Hispanic Asian/Pacific Islander; NHAIAN, Non‐Hispanic American Indian/Alaska Native.

^a^ Data are presented as death numbers followed by percentages of the total deaths of gallbladder cancer in the database.

^b^ Time‐trends were computed using Joinpoint Regression Program (v4.9.0.1, NCI) with three maximum joinpoints allowed (4‐line segments).

### 3.3 Sensitivity analysis

Our sensitivity analysis of overall incidence and mortality rates of GBC after excluding the calendar year 2020 showed largely similar results to the initial analysis (Tables 3 and 4).

**TABLE 3:** Sensitivity analysis of the time‐trends for gallbladder cancer incidence rates among different race and ethnic groups categorized by tumor stage at diagnosis.

Race/Ethnic Group	Cases (*N* = 72,835)^a^	Trends^b^				
		Time period	APC (95% CI)	*p*‐Value	AAPC (95% CI)	*p*‐Value
All stages						
NHW	46,516 (63.86%)	2001–2012	−0.56 (−0.94 to −0.18)	0.007	−0.95 (−1.28 to −0.61)	<0.001
		2012–2019	−1.55 (−3.51 to −1.02)	<0.001		
NHB	9988 (13.71%)	2001–2019	1.42 (0.94 to 1.91)	< 0.001	1.42 (0.94 to 1.91)	<0.001
Hispanic	10,390 (14.27%)	2001–2019	−1.51 (−1.91 to −1.10)	<0.001	−1.51 (−1.91 to −1.10)	<0.001
NHAPI	3416 (4.69%)	2001–2019	−0.91 (−1.80 to −0.02)	0.04	−0.91 (−1.80 to −0.02)	0.04
NHAIAN	744 (1.02%)	2001–2019	−2.90 (−4.92 to −0.84)	0.004	−2.90 (−4.68 to −1.07)	0.004

Abbreviations: NHW, Non‐Hispanic White; NHB, Non‐Hispanic Black; NHAPI, Non‐Hispanic Asian/Pacific Islander; NHAIAN, Non‐Hispanic American Indian/Alaska Native.

^a^ Data are presented as death numbers followed by percentages of the total deaths of gallbladder cancer in the database.

^b^ Time‐trends were computed using Joinpoint Regression Program (v4.9.0.1, NCI) with 3 maximum joinpoints allowed (4‐line segments).

**TABLE 4:** Sensitivity analysis of the time‐trends for gallbladder cancer mortality rates among different race and ethnic groups.

Race/Ethnic group	Deaths (*N* = 41,148)^a^	Trends^b^				
		Time period	APC (95% CI)	*p*‐Value	AAPC (95% CI)	*p*‐Value
All stages						
NHW	28,590 (69.48%)	2000–2017	−1.55 (−1.82 to −1.28)	<0.001	−2.03 (−2.83 to −1.22)	<0.001
		2017–2019	−5.97 (−13.34 to 2.02)	0.12		
NHB	5337 (12.97%)	2000–2019	0.32 (−0.28 to 0.91)	0.27	0.32 (−0.28 to 0.91)	0.27
Hispanic	4912 (11.94%)	2000–2019	−1.99 (−2.62 to −1.35)	<0.001	−1.99 (−2.62 to −1.35)	<0.001
NHAPI	1834 (4.46%)	2000–2019	−1.07 (−2.00 to −0.13)	0.02	−1.07 (−2.00 to −0.13)	0.02
NHAIAN	397 (0.96%)	2000–2019	−3.82 (−5.43 to −2.18)	<0.001	−3.82 (−5.43 to −2.18)	<0.001

Abbreviations: NHW, Non‐Hispanic White; NHB, Non‐Hispanic Black; NHAPI, Non‐Hispanic Asian/Pacific Islander; NHAIAN, Non‐Hispanic American Indian/Alaska Native.

^a^ Data are presented as death numbers followed by percentages of the total deaths of gallbladder cancer in the database.

^b^ Time‐trends were computed using Joinpoint Regression Program (v4.9.0.1, NCI) with three maximum joinpoints allowed (4‐line segments).

## 4 DISCUSSION

Nationwide data evaluating approximately 98% of GBC patients in the US show that GBC incidence trends have been decreasing in all race and ethnic groups except NHB who experienced a significant increase in incidence between 2001 and 2014 and plateauing afterward. This increase is driven by tumors diagnosed at a distant stage. Furthermore, we show that mortality rates of GBC were decreasing in NHW, Hispanic, NHAPI, and NHAIAN populations while remaining stable in NHB.

A growing body of literature has shown that there are racial and ethnic disparities in the incidence rates of GBC. A nationwide database analysis in the United States showed that the highest incidence of GBC occurred in Korean men in Los Angeles and American Indian women in New Mexico from 1998 to 2002.^12^ While in men, American Indians had the second highest incidence of GBC, in women, Hispanic Whites had the second highest incidence.^12^ While the reasons for those findings were unclear, it was hypothesized that either genetic or environmental factors contribute to this pattern in non‐White ethnic groups.^12^ Another study provided analysis of the GLOBOCAN 2018 data, a database providing comprehensive international estimates of incidence and mortality in 185 countries, showed that the incidence rate of GBC in the United States was highest among American Indians and Alaskan Native people.^3^ The study also revealed that the incidence of GBC in the United States has been decreasing from 1975 to 2015, in all racial and ethnic groups, with the exception of NHB. Additionally, another analysis of the SEER database between 2001 and 2012 showed that GBC incidence rates were either stable or decreasing in all races and ethnicities, with the exception of Blacks who experienced an increase in the incidence.^13^ It was also observed that all races and ethnicities had improvements in survival rates over time, except for Blacks and Hispanics.^13^ Our study provides further evidence showing racial and ethnic variations in GBC incidence trends. We demonstrate that NHB experienced a significant increase in GBC incidence between 2001 and 2014, followed by a plateauing of the rates. Furthermore, our study also examined the incidence trends of GBC in NHAPI, which were not extensively studied in previous literature, showing decreasing incidence rates in this population between 2001 and 2020. When looking at the average change in incidence during the last two decades among different populations, we conclude that GBC incidence rates have been improving more in all racial and ethnic groups compared to NHB.

There is sparse literature on the differences in the incidence of GBC per stage at diagnosis. A prior analysis of the SEER database from 2000 to 2013 evaluating 7507 patients diagnosed with GBC found no racial/ethnic disparities in the stage of disease, classified as early and advanced, at presentation.^14^ Another analysis of 18,124 patients with GBC between 1973 and 2009 found that Asian/Pacific Islanders and Hispanics had a higher proportion of cases diagnosed at a localized stage.^15^ Our study provides a comprehensive analysis of race/ethnic‐specific GBC incidence rates stratified by stage at diagnosis. We demonstrate that there were significant differences, with NHB having an increased incidence of late‐stage GBC, while Hispanics having decreasing incidence of late‐stage disease.

While there are also significant differences in mortality from GBC based on racial and ethnic group, the literature is inconsistent. A nationwide analysis of the SEER database from 1992 to 2006 suggests that mortality rates were decreasing in Hispanics and Asian/Pacific Islanders, whereas there was no change with African‐Americans and non‐Hispanic Whites.^12^ Another population‐based study in the US from 1973 to 2009 suggested that survival was better in Asian/Pacific Islanders, compared to all other racial groups. This study also found that patients who received both radiation and surgery had the highest survival rates.^15^ Another population‐based review of biliary tract cancers in the United States found that the highest rate of GBC‐related mortality occurred in Hispanics and NHAIAN.^16^ Our study adds to the literature by providing race/ethnic‐specific mortality trends in a significantly large sample size over a recent time‐period, showing that mortality rates decreased in NHW, Hispanics, NHAPI, and NHAIAN, but remained stable in NHB.

While the exact causes for the increasing incidence and no improvement in mortality rates of GBC in NHB are unclear, there might be possible explanations. GBC is rare, presents with non‐specific symptoms, and lacks widely accepted screening modalities. Some of the risk factors associated with increased risk of GBC include personal or family history of gallstones and other factors linked to gallstone formation, such as female sex, parity, a carbohydrate‐rich diet, and obesity.^17^, ^18^ Although there is a paucity of studies on the development of GBC in NHB, health disparities might be contributing to this finding. African‐Americans have higher rates of obesity, diabetes mellitus, insulin resistance, and hyperinsulinemia, compared to their White counterparts.^19^, ^20^ Furthermore, due to socioeconomic factors, NHB lack adequate access to quality healthcare and can experience bias in the healthcare system, which can lead to disparities in early diagnosis and survival among this racial/ethnic group.^21^ A prior retrospective study of 1554 patients who required cholecystectomy showed that non‐White populations were more likely to present emergently and require an open procedure.^4^ This may be due to the fact that minority patients may delay seeking treatment of gallbladder pathology which can lead to more advanced disease and subsequently put them at risk of developing GBC. NHB tend to be either uninsured or have Medicaid compared to other races/ethnicities, which can possibly explain the increased incidence of GBC detected at a later stage.^22^ Another avenue that needs further investigation is the racial disparities in the rates of cholecystectomy. A prior nationwide analysis showed that African Americans and Asian/Pacific Islanders had lower rates of cholecystectomies compared to Whites.^23^ Our study also showed that mortality rates remained stable in NHB, while decreasing in all other groups. A study examining SEER data showed that Black and Hispanic populations were less likely to undergo surgery for gallbladder cancer compared to Whites.^14^ Another SEER analysis of patients with potentially respectable pancreatic adenocarcinoma found that Blacks were less likely to be referred for surgery compared to whites, suggesting that there may be bias from the perspective of the healthcare provider,^24^ and this might be playing a role in our findings of no improvement in mortality rates. Our data, along with previous literature, suggests that there are multilevel factors at play in the healthcare system, which could explain our significant findings in the NHB population. Our findings shed light on the racial and ethnic disparities in the diagnosis and outcomes of GBC in the US. This is essential in guiding health policies which can help in the implementation of targeted healthcare interventions that are tailored toward specific populations with the aim of bridging the inequalities between different US populations.

Some of the strengths of our study include the large sample size (76,873 patients and 43,411 deaths attributed to GBC) over a recent time period (2001–2020 for incidence and 2000–2020 for mortality). Furthermore, we utilized joinpoint regression for time‐trend analysis which is usually recommended for cancer epidemiology studies in such large databases.^25^ Additionally, we provide a comprehensive analysis of GBC incidence and mortality rates stratified by race and ethnicity and stage at diagnosis, offering further insight into recent trends in the US. However, our study suffers from several limitations. One of the major limitations includes the lack of clinical variables to assess risk factors associated with the occurrence and outcomes of GBC in different race/ethnic populations. However, our study is observational and hypothesis‐generating, aiming to highlight recent trends and guide policy makers and future research toward further investigation of the findings. Other limitations include the ones that can arise in large databases such as the possibility of records loss and miscoding in the SEER database,^26^ and with that in mind, these can also apply to USCS and NCHS databases given that they obtain their data in similar method to SEER. Having said that, the USCS and NCHS are the most compressive databases on incidence and mortality, respectively, and undergo rigorous quality checks before the publication of their data.^8^, ^9^

## 5 CONCLUSION

Nationwide data in the US, covering nearly all of the population over the last two decades, suggest that minority patients of NHB have been experiencing an increase in GBC incidence between 2001 and 2014 with plateauing afterward, compared to other race and ethnic groups who experienced a decline in the incidence. This increase in NHB is mostly due to late‐stage tumors, and is mostly driven by the first decade followed by stabilization in the second decade. We also show that NHB have also been lacking an improvement in GBC mortality, compared to other race and ethnic groups who experienced decreasing mortality. While the reasons behind these findings are unclear, it can be due to a lack of timely access to healthcare leading to delayed diagnosis and worse outcomes. Future studies are warranted to investigate contributions to the revealed racial and ethnic disparities in GBC incidence, especially the increasing trend of late‐stage GBC in NHB, with the goal of improving early detection and decreasing mortality.

## AUTHOR CONTRIBUTIONS

**Yazan Abboud:** Conceptualization (lead); data curation (lead); formal analysis (lead); investigation (lead); methodology (lead); resources (lead); software (lead); visualization (lead); writing – original draft (lead). **Lawanya Singh:** Writing – original draft (supporting). **Madison Fraser:** Writing – original draft (supporting). **Chun‐Wei Pan:** Writing – review and editing (equal). **Ibrahim Abboud:** Writing – review and editing (equal). **Islam H. Mohamed:** Writing – review and editing (equal). **David Kim:** Writing – review and editing (equal). **Saqr Alsakarneh:** Writing – review and editing (equal). **Fouad Jaber:** Writing – review and editing (equal). **Benjamin Richter:** Writing – review and editing (equal). **Ahmed Al‐Khazraji:** Writing – review and editing (equal). **Kaveh Hajifathalian:** Writing – review and editing (equal). **Sima Vossough‐Teehan:** Writing – review and editing (lead).

## CONFLICT OF INTEREST STATEMENT

The authors have no conflict of interest to declare.

## ETHICS STATEMENT

Data used in this study are de‐identified and publicly available and thus were exempted from IRB review.

## Data Availability

Data used in this study are de‐identified and publicly available.
